# Desmin and dystrophin abnormalities in upper airway muscles of snorers and patients with sleep apnea

**DOI:** 10.1186/s12931-019-0999-9

**Published:** 2019-02-14

**Authors:** Farhan Shah, Karl A. Franklin, Thorbjörn Holmlund, Eva Levring Jäghagen, Diana Berggren, Sture Forsgren, Per Stål

**Affiliations:** 1 Department of Integrative Medical Biology, Laboratory of Muscle Biology, Umeå University, SE-901 87 Umeå, Sweden; 2 Department of Surgical and Perioperative Sciences, Surgery, Umeå University, Umeå, Sweden; 3 Department of Clinical Sciences, Otolaryngology, Umeå University, Umeå, Sweden; 4 Department of Odontology, Oral and Maxillofacial Radiology, Umeå University, Umeå, Sweden

**Keywords:** Muscle, Upper airway dysfunction, Dysphagia, Pathophysiology, Cytoskeletal abnormalities, desmin, dystrophin

## Abstract

**Background:**

The pathophysiology of obstruction and swallowing dysfunction in snores and sleep apnea patients remains unclear. Neuropathy and to some extent myopathy have been suggested as contributing causes. Recently we reported an absence and an abnormal isoform of two cytoskeletal proteins, desmin, and dystrophin, in upper airway muscles of healthy humans. These cytoskeletal proteins are considered vital for muscle function. We aimed to investigate for muscle cytoskeletal abnormalities in upper airways and its association with swallowing dysfunction and severity of sleep apnea.

**Methods:**

Cytoskeletal proteins desmin and dystrophin were morphologically evaluated in the uvula muscle of 22 patients undergoing soft palate surgery due to snoring and sleep apnea and in 10 healthy controls. The muscles were analysed with immunohistochemical methods, and swallowing function was assessed using videoradiography.

**Results:**

Desmin displayed a disorganized pattern in 21 ± 13% of the muscle fibres in patients, while these fibers were not present in controls. Muscle fibres lacking desmin were present in both patients and controls, but the proportion was higher in patients (25 ± 12% vs. 14 ± 7%, *p* = 0.009). The overall desmin abnormalities were significantly more frequent in patients than in controls (46 ± 18% vs. 14 ± 7%, *p* < 0.001). In patients, the C-terminus of the dystrophin molecule was absent in 19 ± 18% of the desmin-abnormal muscle fibres. Patients with swallowing dysfunction had 55 ± 10% desmin-abnormal muscle fibres vs. 22 ± 6% in patients without swallowing dysfunction, *p* = 0.002.

**Conclusion:**

Cytoskeletal abnormalities in soft palate muscles most likely contribute to pharyngeal dysfunction in snorers and sleep apnea patients. Plausible causes for the presence of these abnormalities is traumatic snoring vibrations, tissue stretch or muscle overload.

## Background

Obstructive sleep apnea is a highly prevalent disorder associated with adverse health consequences [1]. The disorder is characterized by repetitive narrowing and collapse of the upper airways, with snoring and subsequent hypoxia [2]. Inadequate dilating muscle forces are suggested as an important factor in the pathophysiology of obstructive sleep apnea. Nerve injuries due to traumatic snoring vibration have been proposed as one of the causes for the inability of the upper airway muscles to maintain patency during sleep and for the commonly occurring swallowing dysfunction in snorers and sleep apnea patients [3–14]. Although evidence of upper airway neuropathy exists in snorer and sleep apnea patients, acquired muscle injuries as a contributing cause for muscle weakness and pharyngeal dysfunction has gained less attention [11].

We have earlier reported that a small subpopulation of fibres in soft palate muscles of healthy humans lacked or had a truncated form of two cytoskeletal proteins, desmin, and dystrophin [15]. This is specifically interesting since these two cytoskeletal filaments are considered to be ubiquitous and vital for muscle function [16]. Desmin is the major intermediate filament (IF) in mature muscles [16]. It is located at the periphery of the Z-disc and links the entire contractile apparatus to the subsarcolemmal cytoskeleton, the cell nuclei and to other organelles such as mitochondria [17–19]. The network of desmin filaments supports the structural and mechanical integrity of the muscle cell during contraction and contributes to force transmission and load bearing [16]. Dystrophin is localized in the inner part of the sarcolemma where it binds other cytoskeletal protein filaments in the muscle cell to the surrounding extracellular matrix (ECM) through the cell membrane [20]. The C-terminus domain of the dystrophin molecule is associated with the membrane-spanning dystrophin-associated protein complex (DAPC), whereas its N-terminus interacts with actin filaments. The DAPC has significant roles in stabilizing sarcolemma and transmitting force generated in the muscle sarcomere to ECM [21]. In genetic myopathies and animal gene knockout experiments, the absence of these proteins leads to progressive muscle weakness [22, 23].

Presence of abnormalities in cytoskeletal proteins in upper-airway muscles of snoring and sleep apnea patients and its impact on pharyngeal function has not been investigated. Therefore, we aimed to investigate for desmin and dystrophin abnormalities in soft palate muscles of snoring and sleep apnea patients and to evaluate whether these abnormalities relate to deviations in swallowing function and severity of obstructive sleep apnea.

## Methods

### Patients and controls

Twenty-two consecutive patients (1 female, 21 males) referred for upper-airway surgery because of snoring and sleep apnea were included. The exclusion criteria were smoking, previous palatal surgery, systemic disease, medications, and drug abuse. The mean age was 45 years (range 29–60), and the mean body mass index (BMI) was 28 kg/m^2^ (range 21–34). Ten voluntary controls, all males, mean age 38 years (range 30–51) and mean BMI 24 kg/m^2^ (range 22–30), were recruited through advertisements. The exclusion criteria were similar as in patients, but also included habitual snoring and sleep apnea. For reference, a biopsy from an arm muscle, biceps brachii, and a thigh muscle, vastus lateralis, were acquired from two healthy adult male subjects.

### Sleep apnea recordings

All patients and voluntary controls underwent ambulatory overnight sleep apnea recordings (Embletta, Embla systems, Kanata, Canada) using nasal cannula pressure, thoracic and abdominal respiratory effort, finger oximetry (Nonin Oximeter, Plymouth) and a body-positioning sensor. All the recordings were scored manually according to the American Academy of Sleep Medicine recommendations. The definition of an apnea was a ≥ 90% cessation of airflow lasting at least 10 s, while a hypopnea was defined as 50% reduction in airflow compared with baseline, in combination with an oxygen desaturation of ≥3% [24].

### Swallowing examination

Swallowing function was investigated in all patients and voluntary controls using a videoradiographic examination (C-arm, Philips BV 29, field width 23 cm) in an upright position and with lateral and frontal projections. The subjects first swallowed a chewed solid bolus of crisp bread and barium sulphate (Mixobar Esophagus; Astra) and then a liquid barium sulphate contrast bolus (Mixobar High Density; Astra). All standard boluses were repeated twice in each projection. The examinations were evaluated at full speed and slow motion by two investigators blinded for the clinical findings of the subjects. Swallowing function was graded as 1. normal function, 2. mild dysfunction in the presence of one of the following deviant features; premature leakage, velar dysfunction, residual or laryngeal penetration, 3. moderate dysfunction with two or more deviant features in grade 2 or dysfunction of the upper esophageal sphincter, the epiglottis or the propagation wave and, 4. severe dysfunction with aspiration below the vocal cords [25].

### Tissue samples and immunohistochemistry

In patients, the entire base of the uvula was resected in connection with soft-palate surgery. In 3 of the cases, parts of the palatopharyngeus muscle were also available. The samples from voluntary controls were acquired from the corresponding site by using punch biopsy technique, except in one case where complete surgical resection of the uvula was performed. Since a punch biopsy represents only a part of the muscle cross-section, autopsies were acquired from the entire base of uvula from 5 subjects who died accidentally (2 males and 3 females), mean age 54 years (range 46–75), mean BMI 25 kg/m^2^ (range 21–31). The autopsies were used only as a cross-reference to determine that the punch biopsies were representative for the entire muscle cross-section. The exclusion criteria were similar to those of voluntary controls. No medical history of snoring and sleep apnea were reported, and all subjects had a normal craniofacial and oro-pharyngeal anatomy. All samples were taken within 12–24 h post-mortem, a delay that not affects muscle morphology, muscle proteome and fibre typing [26, 27].

The muscle samples were cut into small pieces and oriented for both cross- and longitudinal sectioning. Some samples were immediately mounted in OCT (optimum cutting temperature) compound (Tissue Tek, Miles, Elkhart, IN, USA) and frozen in liquid propane chilled with liquid nitrogen, while others were fixed before freezing using 4% formaldehyde in 0.1 M phosphate buffer, pH 7.0, for 24 h at 4 °C and overnight washing at 4 °C in Tyrodes solution containing 10% sucrose. Five μm thick serial muscle sections were cut in a cryostat and mounted on glass slides. The sections were immunostained with previously characterized monoclonal (mAb) and polyclonal (pAb) antibodies directed against IF desmin, alpha-actinin (a major component of the Z-disk), membrane-associated proteins dystrophin, utrophin, and laminin, and isoforms of contractile myosin heavy chain (MyHC) motor proteins were used (Table 1). A mounting medium with DAPI (4′, 6-diamidino-2-phenylindole dihydrochloride) was used to visualize nuclei (H-1500, Vector Lab, Burlingame, CA, USA). Details of the used multi-staining technique have been described previously [28].

**Table 1:** Antibodies used for immunohistochemistry

Antibody	Product Code	Specificity	Gene^a^	Host/Clone	Dilution	Source
Desmin	M0760	Human and animal desmin	DES	mAb-mouse/ D33	1:100	1
Desmin	18–0016	Human desmin	DES	mAb-mouse/ ZC18	1:1000	2
Desmin	ab15200	Human and animal desmin	DES	pAb-rabbit	1:2000	3
Dystrophin	GTX15277	Human dystrophin (C-terminus)	DMD	pAb-rabbit	1:7500	4
Dystrophin	NCL-DYS1	Human dystrophin (Rod domain)	DMD	mAb-mouse/ Dy4/6D3	1:5	5
Dystrophin	NCL-DYS2	Human dystrophin (C-terminus)	DMD	mAb-mouse/ Dy8/6C5	1:10	5
Dystrophin	NCL-DYS3	Human dystrophin (N terminus)	DMD	mAb-mouse/ DY10/12B2	1:10	5
Alpha-actinin	A7732	Human and animal α-actinin (Sarcomeric)	ACTN2	mAb-mouse/ EA-53	1:500	6
Laminin	PC 128	Human laminin	LAM	pAb-sheep	1:15000	7
Utrophin	sc-33700	Human utrophin	UTRN	mAb-mouse/ 8A4	1:200	8
Slow MyHC	A4.840	Human and animal MyHCI	MyH7	mAb-mouse	1:400	9
Fast A MyHC	A4.74	Human and animal MyHCIIa	MyH2	mAb-mouse	1:500	9

^a^Official gene nomenclature according to OMIM. (http://www.ncbi.nlm.nih.gov/omim). 1. Dako, Sweden, 2. Invitrogen Corporation, CA, USA; 3. Abcam, UK, 4. GeneTex Inc., Taiwan, 5. Novocastra Laboratories Ltd., UK, 6. Sigma-Aldrich Co, UK; 7. Binding site Inc., USA; 8. Santa Cruz Biotechnology Inc. UK, 9. Developmental Studies Hybridoma Bank, developed under the auspices of the NICHD and maintained by the University of Biological Sciences, Iowa City, Iowa, USA

### Muscle fibre type classification

Based on the immunostaining pattern for the different MyHC mAbs, the muscle fibres were classified as slow contracting MyHC-I (type I) or fast contracting MyHC-II (type II) fibres.

### Quantitative and statistical analysis

Muscle cross-sections from uvula of all the patients and controls were included in the quantification. Palatopharyngeus muscles were not included in the quantitative analysis, due to the small sample size and lack of control biopsies. In uvula, 4 to 5 random areas from each muscle cross-section were scanned at 20x magnification with a fluorescence microscope (Leica DM6000B, Leica Microsystems CMS GmbH, Wetzlar, Germany) equipped with a digital high-speed fluorescence charge-coupled device (CCD) camera (Leica DFC360 FX). The number of unstained, weakly stained, or disorderly stained fibres for the Abs directed against desmin and dystrophin were quantified manually on each photo (Photoshop CS5, version 12.0.4, San Jose, CA, USA). The investigators were blinded to the origin of the samples. Comparisons between the mean values of changes in muscle fibres between the two groups were made using the Mann-Whitney U test. For comparing more than two groups, planned one-way ANOVA (analysis of variance) with bootstrapping was used. Both statistical tests do not require an assumption of normality in the distribution of data. Values are presented as the mean ± standard deviation. Results were considered significant at a *p*-value of ≤0.05. All the tests were performed with SPSS (statistical package for social sciences) software (IBM SPSS 23, statistical software, Armok, NY: IBM corp., USA).

## Results

All 22 patients snored, and 14 had obstructive sleep apnea (mean AHI 24, range 5–84). Ten patients had a moderate swallowing dysfunction, 6 patients had a mild dysfunction and 6 had a normal function. None of the 10 voluntary controls snored or had sleep apnea and they all displayed a normal swallowing function.

### Muscle morphology

**Fig. 1:**
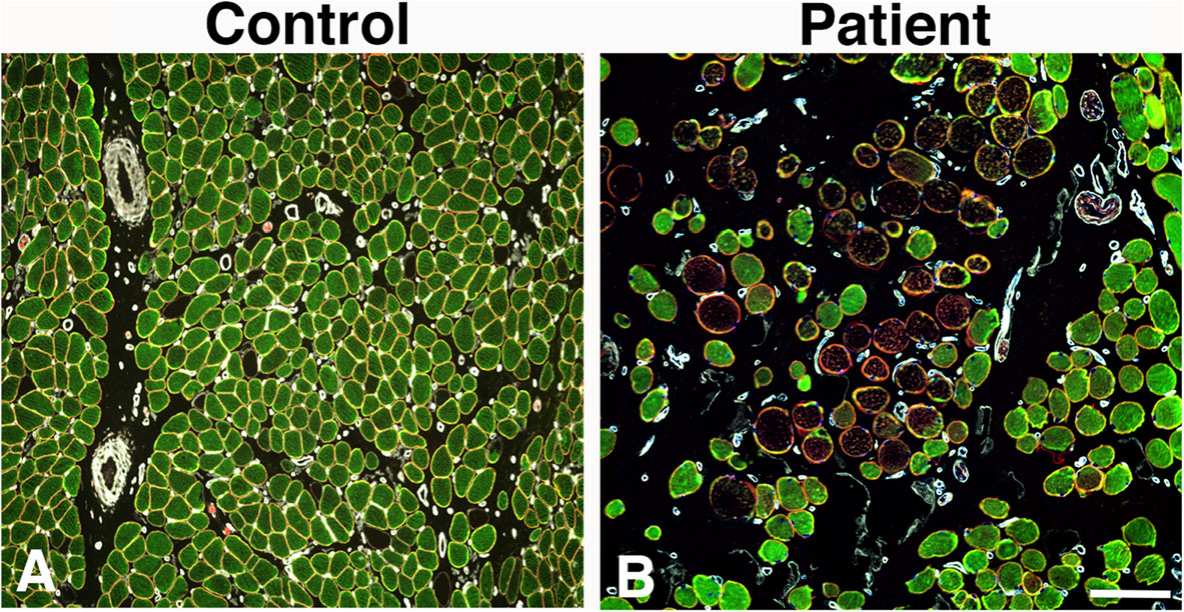
Overview of muscle cross-sections from a control (**a**) and a patient (**b**) immunostained for desmin, dystrophin, and laminin (Desmin, green color, dystrophin, red color, laminin, white color, merged dystrophin and laminin, yellow color). Note the high fibre size variability, high amount of connective tissue and a large number of fibres unstained for desmin in the patient (**b**) compared to the control (**a**). Scale bar 100 μm

The muscle morphology of patients differed distinctly from that of the controls, by having considerably larger variability in muscle fibre size and fibre form, more loosely packed fibres, and a greater amount of connective and fat tissue (Fig. 1).

### Immunoreactivity for cytoskeletal and membrane proteins

**Fig. 2:**
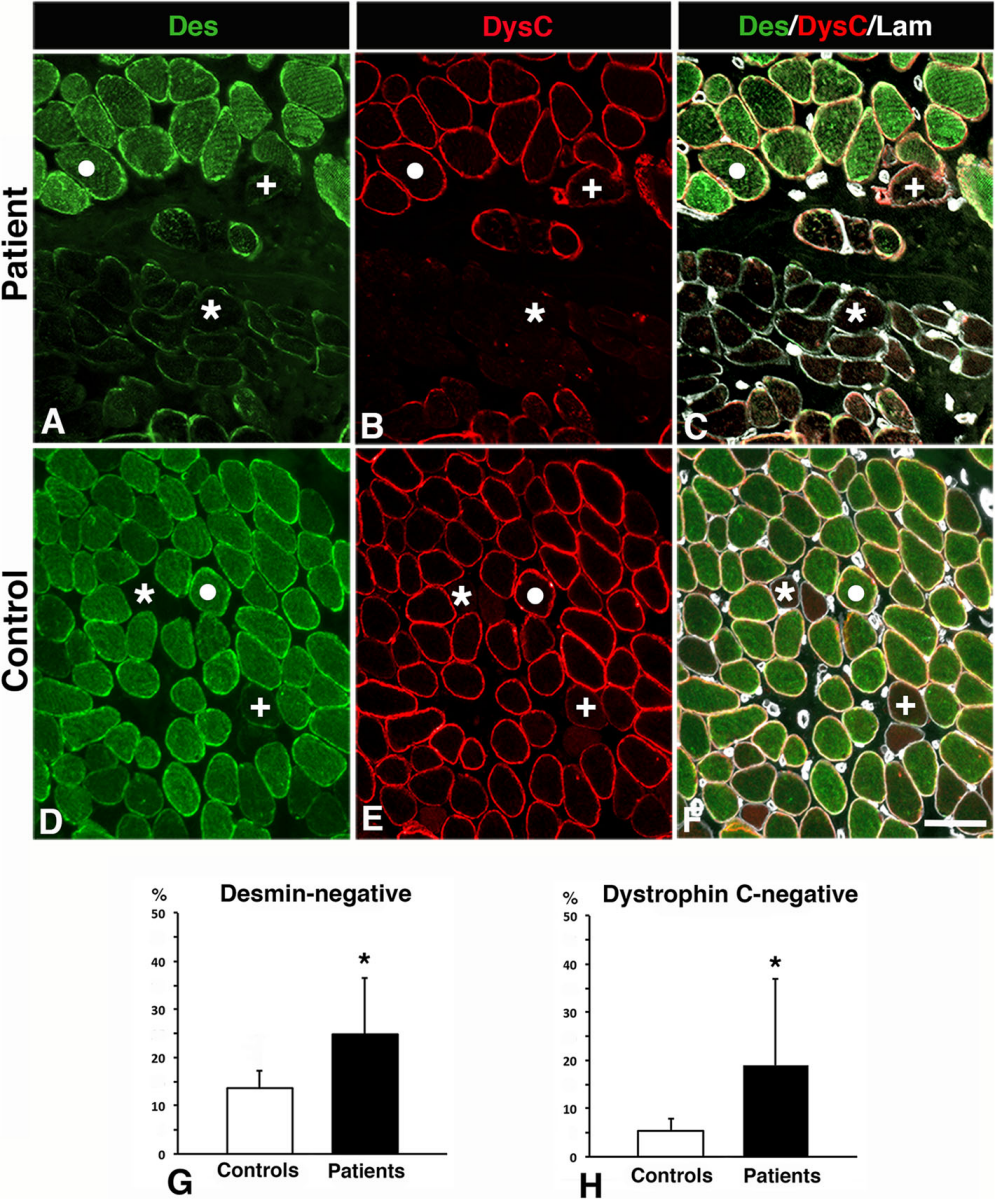
Muscle cross-sections from a patient (**a-c**) and a control (**d-f**) immunostained for desmin (Des, green colour, **a, d**), dystrophin C-terminus (DysC, red colour, **b, e**) and laminin (Lam, white colour, **c, f**). Panels C and F show merged staining. Normal expression of desmin and dystrophin (white dot), desmin-negative fibres (+) and desmin and dystrophin C-terminus-negative fibres (*). Scale bar 50 μm. Graphs **g** and **h** shows the percentage of desmin and dystrophin C-terminus negative fibres in controls and patients (mean and SD). A significant difference (*p* < 0.05) is marked (*)

**Fig. 3:**
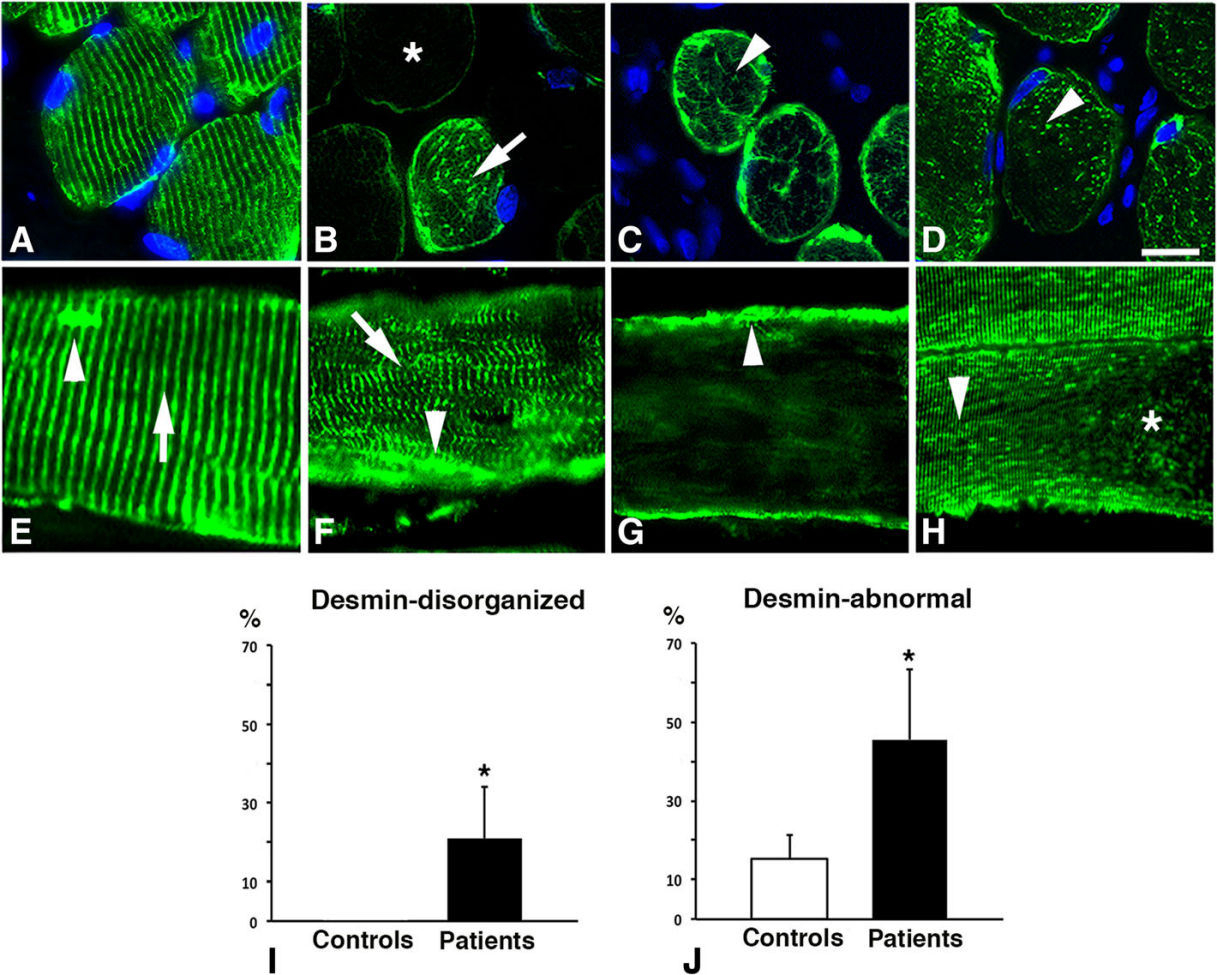
Muscle cross-sections (**a-d**) and longitudinal sections (**e-h**) stained for desmin and nuclei (DAPI) in the uvula muscle of patients. Normal immunoreaction for desmin is shown in (**a**) and abnormal immunoreaction patterns are shown in (**b-h**). Panel **b** shows a desmin-negative fibre (*) and a fibre with desmin distributed as larger dots or striations (arrow). Panel **c** displays an irregular or trabecular pattern of desmin (arrowhead) and panel **d** shows desmin evenly distributed as fine grainy dots (arrowhead). Panel **e** shows a longitudinally sectioned muscle fibre with a normal striated pattern of desmin in the Z-line (arrow) and subsarcolemmal aggregation of desmin (arrowhead). Panel **f** displays myofibrillar disorganization (arrow) and desmin aggregates (arrowhead), Panel **g** shows a desmin-negative fibre with dense subsarcolemmal staining (arrowhead) and panel **h** shows an area with a striated and punctuated staining pattern for desmin (arrowhead) and an area lacking desmin (*). Scale bar **a-h**, 25 μm. Bar graphs showing the percentage of fibres with disorganized desmin (**i**) and the pooled values of desmin-negative and desmin-disorganized fibres (**j**, desmin-abnormal) in controls and patients (mean and SD). A significant difference (p < 0.05) is marked (*)

Two major abnormal immunostaining patterns for desmin were observed in both small and large sized fibers in uvula and palatopharyngeus muscle cross-sections of patients. One of the patterns, observed both in controls and patients, displayed lack of immunoreactivity for desmin, here referred to as desmin-negative fibres (Figs. 1, 2, 3). The other immunoreaction pattern, exclusively present in muscle fibres of patients, displayed a disorganization of desmin ranging from fibres expressing small to large aggregates to fibres with an extensive derangement of desmin in a lobulated or trabecular pattern (Fig. 3). These fibres are referred to as desmin-disorganized fibres. A weak to intense subsarcolemmal staining for desmin was often present in both desmin-negative and desmin-disorganized fibres (Figs. 2 and 3).

In longitudinal muscle sections of patients, some of the muscle fibres showed disruptions in the normal striations of desmin in the Z-band region of the sarcomere, while others displayed complete disorganization or absence of desmin (Fig. 3 e-h). The disorganization or absence of desmin was in some fibres only located in specific regions along the length of the myofibril. Aggregates of desmin spanning over several Z-disks were often observed within the myofibrils (Fig. 3 e-h). In controls, all muscle fibres expressing desmin exhibited a normal distribution of desmin along the Z-disk, seen as horizontal striations of labeling (Fig. 3a). The immunoreaction patterns observed in both patients and controls were confirmed with all Abs directed against desmin (Table 1).

**Fig. 4:**
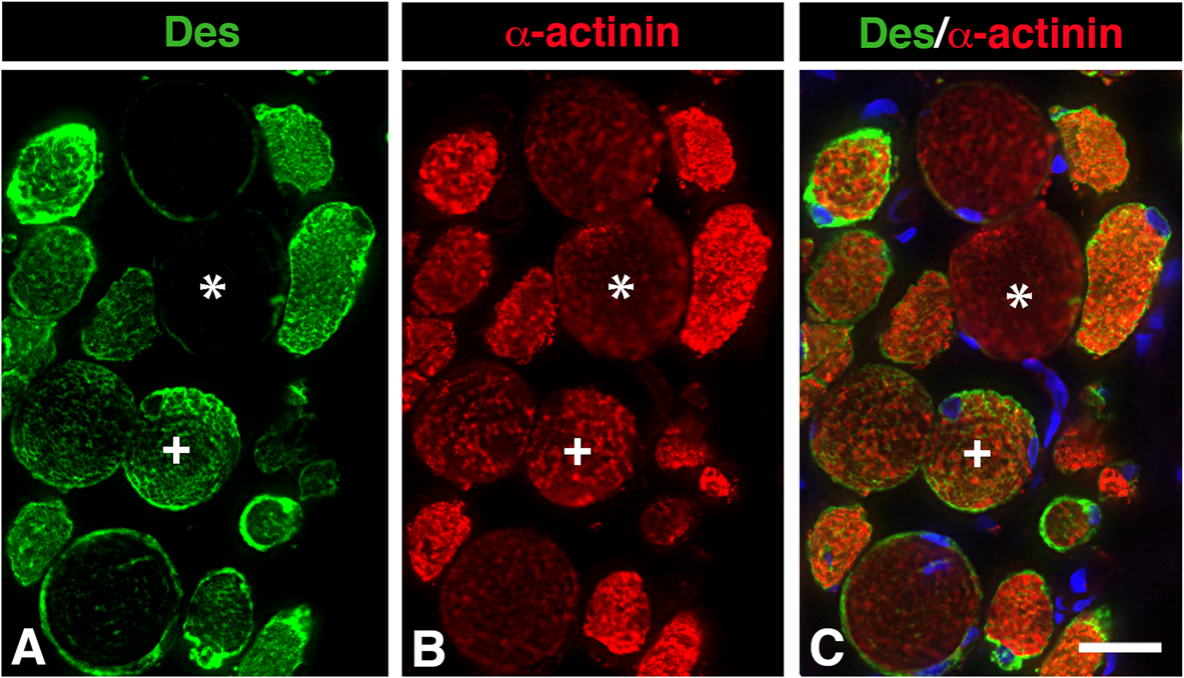
Muscle cross-section from the uvula of a patient immunostained for desmin (Des, green color, **a**) and alpha-actinin (α-actinin, red color, **b**). Panels **c** shows merged staining for desmin, alpha-actinin, and nuclei (DAPI). A desmin negative fibre weakly stained for alpha-actinin (*) and a fibre with immunoreaction for both desmin and alpha-actinin (+). Note the large variability in staining pattern and intensity for both desmin and alpha-actinin. Scale bar 25 μm

In control sections where desmin was co-labeled with alpha-actinin, a unique marker for the Z-disc, an intact sarcomeric structure were observed in all muscles, irrespectively of absence or presence of immunoreaction for desmin. In patients, the immunoreaction for alpha-actinin in desmin negative fibres was generally weak, while all desmin disorganized fibres showed deranged immunolabeling for alpha-actinin ranging from weak to strong (Fig. 4). Muscle fibres in patients expressing a normal staining pattern for desmin also showed a normal staining pattern for alpha-actinin at the Z-disk.

The immunostaining for the different subunits of the dystrophin molecule revealed that a subgroup of muscle fibres, especially in patients, was unstained or weakly stained for the dystrophin C-terminus (-COOH), while the rod and the N-terminus (-NH_2_) domain of the dystrophin molecule showed normal immunoreactivity in the sarcolemma (Figs. 1 and 2). All muscle fibres lacking immunoreaction for dystrophin C-terminus also displayed abnormal immunoreaction for desmin (Figs. 1 and 2). The cell membrane of all muscle fibres was normally stained for laminin in both patients and controls (Fig. 1). Utrophin, a paralog to dystrophin, was not upregulated in any muscle fibres.

All reference samples from limb muscles showed normal immunoreaction pattern for all antibodies directed against cytoskeletal and membrane proteins.

### Quantification of cytoskeletal abnormalities

The quantification and statistical analyses of fibres with abnormal expression of desmin and dystrophin in the uvula muscle were based on an evaluation of 7836 muscle fibres in patients and 1443 muscle fibres in the biopsies from voluntary controls.

For cross-reference, the biopsies from voluntary controls and patients were compared with the results from control autopsies (*n* = 3453 muscle fibres).

### Proportion of desmin and dystrophin abnormalities

**Fig. 5:**
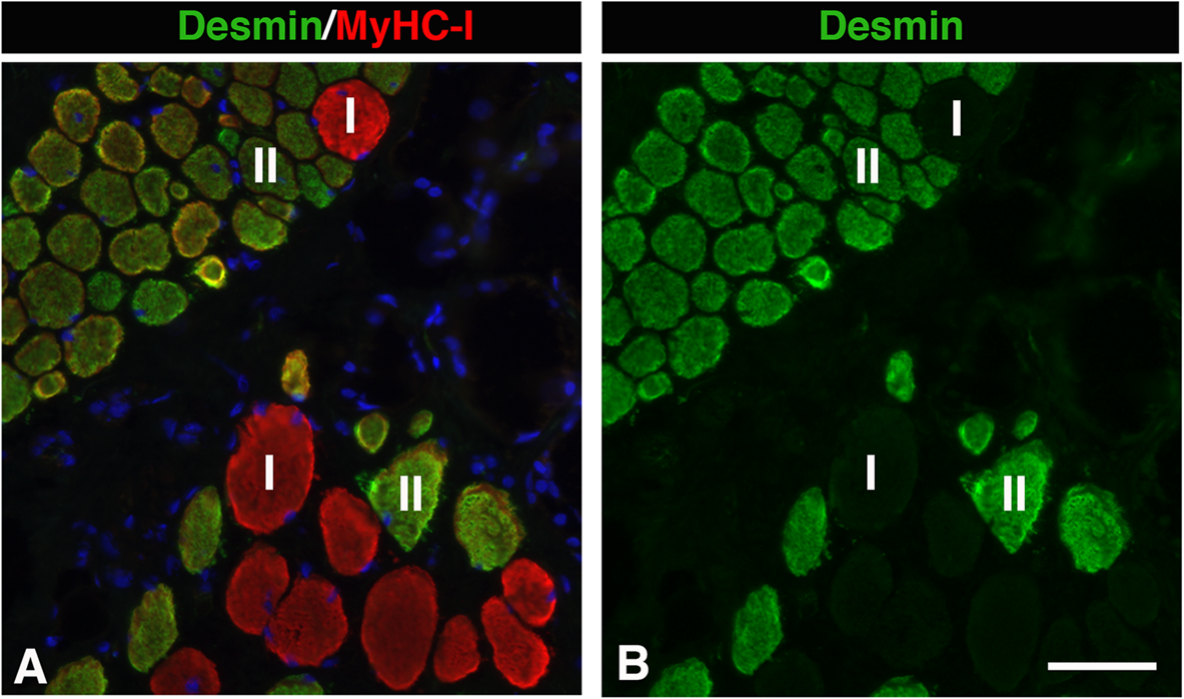
Muscle cross-section from the uvula of a patient stained for desmin and slow contractile protein MyHCI. Panel **a** shows merged staining for desmin and slow MyHCI, while panel **b** shows only desmin. Fibres expressing slow MyHCI (red, I) and fast MyHC II (green, II) are marked. Note that the deficiency of desmin is more common among fibres expressing MyHCI. Scale bar 50 μm

All samples from patients had cytoskeletal abnormalities in the muscle fibre population. The proportion of desmin-disorganized fibres was 21 ± 13% in patients, while they were not present in controls (Fig. 3 i). No specific fibre type predilection was observed for fibres with disorganized desmin. Desmin-negative fibres were found in 25 ± 12% of the fibre population in patients, which was significantly more than in controls (14 ± 7%, *p* = 0.009) (Fig. 2 g). These fibres were predominantly of slow type I (64 ± 22%) (Fig. 5). The pooled fractions of desmin-negative and desmin-disorganized fibres (46 ± 18%), referred to as desmin-abnormal fibres*,* were significantly higher in patients than in controls (*p* < 0.001) (Fig. 3 j). The frequency of dystrophin C-terminus-negative fibres was also significantly higher in patients (19 ± 18%) compared to controls (7 ± 2%, *p* = 0.04) (Fig. 2 h).

### Cross-reference with control autopsies

No significant differences in muscle morphology and proportions of desmin-negative fibres were observed between control autopsies and control biopsies. Desmin disorganized fibres were not observed either in control biopsies or autopsies. The proportion of cytoskeletal abnormalities in patient biopsies was significantly higher compared to control autopsies (data not shown).

### Proportion of desmin and dystrophin abnormalities in patients with and without a swallowing dysfunction

**Fig. 6:**
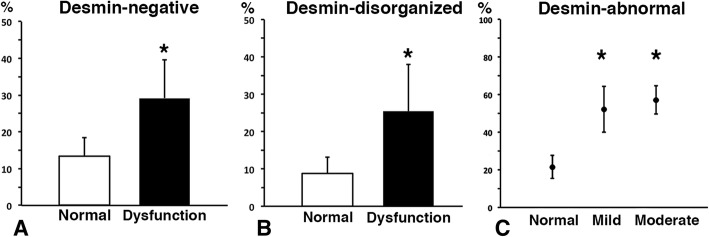
Bar graphs showing the percentage of desmin-negative fibres (**a**) and desmin-disorganized fibres (**b**) in the uvula muscle of patients with normal swallowing or dysfunctional swallowing (mean and SD). Graph (**c**) shows proportion (%) of desmin-abnormal fibres, i.e. pooled percentage of desmin-negative and desmin-disorganized fibres, in patients with normal swallowing function and mild and moderate swallowing dysfunction, respectively (mean and SD). A significant difference (p < 0.05) is marked (*)

Both desmin-negative and desmin-disorganized muscle fibres were significantly higher in patients with swallowing dysfunction compared with patients with normal function (*p* = 0.005) (Fig. 6 a and b).

The pooled fractions of desmin-negative and desmin-disorganized fibres i.e. desmin-abnormal fibres, were 55 ± 10% in patients with a mild to moderate swallowing dysfunction compared to 22 ± 6% in patients with a normal swallowing function, (*p* = 0.002) (Fig. 6 c).

The frequency of dystrophin C-terminus-negative fibres was higher in patients with a swallowing dysfunction than in patients with a normal function (18 ± 13% vs. 7 ± 5%, *p* = 0.007).

### Comparison between cytoskeletal abnormalities and AHI

All snoring and sleep apnea patients had a significantly higher proportion of cytoskeletal abnormalities compared to controls, but the degree of abnormalities did not show any significant relationship with the severity of AHI or oxygen desaturation.

## Discussion

We here report evidence of cytoskeletal abnormalities in soft palate muscles of snoring and sleep apnea patients. A significant number of muscle fibres revealed an absence, derangement or an abnormal isoform of two important cytoskeletal proteins, desmin, and dystrophin. Interestingly, these cytoskeletal abnormalities were significantly more frequent in snoring and sleep apnea patients with swallowing dysfunction than in patients with normal swallowing function. The present findings highlight that apart from previous reports of sensorimotor neuropathy in upper airways [5–10, 29, 30], cytoskeletal abnormalities in muscles have to be taken into account in the pathophysiology of pharyngeal dysfunction in snorers and sleep apnea patients. Any changes rendering the upper-airway muscles less capable of generating force have the potential to affect pharyngeal function.

The high proportion of fibres with disorganized desmin as well as histopathological changes such as atrophic and hypertrophic fibres, fascicular atrophy and fibrosis in the palate muscles of snorer and sleep apnea reflect muscle weakness in the upper airways due to local injury. The disorganization of desmin often resembled the pattern seen in myofibrillar myopathies where the primary clinical feature is progressive muscle weakness [22, 31]. The pathogenesis of these two distinct types of changes seem, however, to differ. While the presence of atrophic fibers is probably due to acquired motor neuropathy secondary to snoring vibratory damage [5, 12–14, 32], the desmin disorganized fibres seems preferably related to myofibrillar injury and muscle overload. This assumption is based on the fact that hypertrophic fibres also displayed disorganized desmin, indicating a frequent use and thus an intact nerve-innervation. Moreover, cytoskeletal disruption has been reported in muscle fibres subjected to overload [33, 34]. Nonetheless, denervation cannot be excluded for the changes of desmin observed in fibres of smaller size. Volodin et al., [35] reported that slow muscle fibre atrophy induced by denervation initially causes phosphorylation and ubiquitination of desmin, followed by dissociation of desmin filaments and protein degradation. However, this process may account for desmin abnormalities in fibres with an atrophic appearance, but not for the abnormalities in the hypertrophic fibres.

Another possible basis for desmin abnormalities is local disturbances in blood circulation in snorers. There is evidence that long-standing exposure to mechanical vibrations in limbs causes vascular damage, reduction in blood flow, mitochondrial disorganization, as well as cytoskeletal derangement and decreased muscle strength [36–38]. Disturbed blood circulation in limb muscles due to peripheral artery disease (PAD) has also been shown to cause an abnormal distribution of desmin as well as an aberrant muscle fibre morphology including irregular and patchy distribution of the mitochondria [39]. Interestingly, this maldistribution of desmin and mitochondria resembles the abnormal derangement of desmin and mitochondrial distribution previously reported in palate muscle fibres of patients [40]. Desmin has a key role in anchoring of mitochondria, and mutations of desmin are reported to substantially disturb spatial orientation and function of mitochondria in muscles [18, 19]. Accordingly, disorganization of desmin and mitochondria, together with our previous findings of reduced muscle capillarization in the soft palate of long-term snorers [41], underpins a disturbed energy production and muscle weakness.

The novel finding of muscle fibres lacking desmin in the soft palate of both patients as well as healthy adult and infants [15], raises interesting questions about their origin. Muscle fibres containing disorganized or disrupted desmin have been described in several myopathies [31], but no disease or neuromuscular injuries have been related to lack of desmin in muscle fibres. Although a transient loss of desmin has been observed in muscles following acute or eccentric activity in animal experiments, the expression of desmin returned to normal levels after a short time [33, 34]. Interestingly, Janbaz et al. [42] reported that a subgroup of fibres in healthy human extraocular muscles lacked or had faint staining for desmin. Extraocular muscles have, as palate muscles, intricate muscle anatomy with several muscles lacking firm attachment at one end and acting against each other in performing precise movements of the eye. In light of this, the presence of fibres lacking desmin can constitute an evolutionary cytoskeletal specialization of the muscle fibres to meet requirements in various oro-pharyngeal functions. Additionally, while desmin disorganized fibres did not have any fibre type predilection, fibres lacking desmin were mainly of slow phenotype I, a finding further strengthening cytoskeletal fibre phenotype specialization. Thus, the significantly higher proportion of fibres with an absence of desmin in muscles from patients compared to healthy controls indicates either a genetic link or that these fibres better survive muscle overload or neuromuscular trauma by snoring and tissue stretch. Since fibres lacking desmin preferentially were of slow type I, the high proportion of desmin negative fibres in snoring and sleep apnea patients could relate to the fact that the motor-nerve damage preferentially affects fibres belonging to type II motor-units, which is in line with some previous studies [7, 12]. The reason why cytoskeletal abnormalities in certain fibres were only observed in specific regions along the length of myofibril needs further investigation.

Another significant finding was that a subpopulation of the desmin-abnormal fibres also lacked immunoreactivity for the C-terminus of dystrophin. The C-terminus domain of the dystrophin molecule is considered to be crucial for normal muscle function, as it binds to the glycoprotein complex in the sarcolemma and transfers forces from the contractile apparatus to the extracellular matrix and adjoining muscle fibres [20, 23]. Physiological studies demonstrated that force production is significantly reduced in muscle fibres showing a deficiency of dystrophin [43]. Moreover, extensive data report that a dystrophin-deficient sarcolemma is fragile and results in increased permeability of membrane-impermeable molecules, especially after physical exercise. In Duchenne muscular dystrophy, loss or truncated forms of dystrophin leave the membrane highly susceptible to contraction-induced injury and hypoxic stress [44], which has deleterious consequences for the intra-myofibrillar environment. Thus, the high proportion of dystrophin C-terminus deficient fibres in snorers and sleep apnea patients might render the muscle more vulnerable to high contraction stress.

Utrophin, a paralog to dystrophin, is expressed on the sarcolemma of developing and regenerating fibres, but it is ultimately replaced by dystrophin in maturing fibres [45]. In Duchenne muscular dystrophy, where dystrophin is lacking, utrophin is strikingly upregulated [46]. However, utrophin was not upregulated in muscle fibres lacking immunoexpression for the dystrophin C-terminus in both patients and controls. Hence, further studies have to rule out whether the truncated form of dystrophin in desmin abnormal fibres is a consequence of local injuries or that the C-terminal of the dystrophin molecule has a specific configuration in soft palate muscles.

Several investigators have proposed that obstructive sleep apnea is a progressive heavy snorers disease [47–49]. Our findings reinforce traumatic snoring vibrations as well as muscle overload as the most credible mechanism for the high presence of fibres with abnormal cytoskeletal distribution in patients. This is strengthened by the observation that alpha-actinin was weakly stained or deranged in most of the desmin abnormal fibres in patients, while all fibres in controls, irrespectively of desmin expression, showed an intact Z disk structure. Additionally, the findings that long-standing exposure to vibrations causes nerve and muscle injuries, including cytoskeletal and mitochondrial disorganization, vascular damage, reduction in blood flow, and decreased muscle strength further support vibratory trauma as a cause for structural muscle changes. Moreover, contraction during lengthening of muscle has been reported to cause histopathological changes in muscle fibres [34], a process that can occur during sleep when the dilator muscles try to counteract the collapsing forces while breathing. Therefore, nerve and muscle injuries, as well as disturbances in blood flow, may affect pharyngeal function in snorers that over time contributes to the development of sleep apnea. Treatment strategies aimed at reducing trauma from snoring and muscle overload, as well as strengthening the upper-airway muscles, could have long-term benefits.

Although the proportion of desmin and dystrophin abnormalities in sleep apnea patients were significantly higher than in controls, no relation to the severity of sleep apnea (AHI) could be established. This reflects the complex interaction of several central and peripheral etiological risk factors in the development and progression of obstructive sleep apnea [50]. Hence, the impact of cytoskeletal abnormalities on upper airway muscle function should be considered together with factors such as the degree of muscle relaxation during sleep, upper airway anatomy, nerve injuries, and other co-morbid conditions in snorers and sleep apnea patients. Future studies with larger cohorts could shed more light on the question if a particular genotype or phenotype of patients exists based on the present findings in snorers and sleep apnea patients.

## Conclusions

To conclude, the present data show that the expression of cytoskeletal proteins in muscle fibres of the soft palate in snoring and sleep apnea patients differ from healthy individuals and from limb muscles. Cytoskeletal abnormalities including absence, disorganization, and accumulation of desmin as well as a truncated form of dystrophin were common in the soft palate muscles of patients, but not in controls. The link between cytoskeletal abnormalities and swallowing dysfunction supports impaired muscle function in the upper airways of patients. Hence, in addition to neuropathy, cytoskeletal myopathy seems to be a contributing factor in the pathophysiology of pharyngeal muscle dysfunction in the upper airways of sleep apnea patients.

## Abbreviations

AHI: Apnea-hypopnea index
ANOVA: Analysis of variance
BMI: Body mass index
CCD: Charged couple device
DAPI: 4′, 6-diamidino-2-phenylindole dihydrochloride
mAb: Monoclonal antibody
MyHC: Myosin heavy chain
OCT: Optimum cutting temperature
pAb: Polyclonal antibody
PAD: Peripheral artery disease
SD: Standard deviation
SPSS: Statistical package for social sciences
